# Visible and Near-Infrared Spectroscopy Enables Differentiation of Normal and Early Osteoarthritic Human Knee Joint Articular Cartilage

**DOI:** 10.1007/s10439-023-03261-7

**Published:** 2023-06-18

**Authors:** Awuniji Linus, Petri Tanska, Jaakko K. Sarin, Ervin Nippolainen, Virpi Tiitu, Janne.T. A. Mäkelä, Juha Töyräs, Rami K. Korhonen, Mika E. Mononen, Isaac O. Afara

**Affiliations:** 1 Department of Technical Physics, University of Eastern Finland, 70211 Kuopio, Finland; 2 Department of Medical Physics, Medical Imaging Center, Pirkanmaa Hospital District, Tampere, Finland; 3 Institute of Biomedicine, University of Eastern Finland, Kuopio, Finland; 4 Science Service Center, Kuopio University Hospital, Kuopio, Finland; 5 School of Information Technology and Electrical Engineering, The University of Queensland, Brisbane, Australia

**Keywords:** Osteoarthritis, Cartilage, Visible light spectroscopy, Near-infrared spectroscopy, Machine learning, Biomechanics

## Abstract

Osteoarthritis degenerates cartilage and impairs joint function. Early intervention opportunities are missed as current diagnostic methods are insensitive to early tissue degeneration. We investigated the capability of visible light-near-infrared spectroscopy (Vis-NIRS) to differentiate normal human cartilage from early osteoarthritic one. Vis-NIRS spectra, biomechanical properties and the state of osteoarthritis (OARSI grade) were quantified from osteochondral samples harvested from different anatomical sites of human cadaver knees. Two support vector machines (SVM) classifiers were developed based on the Vis-NIRS spectra and OARSI scores. The first classifier was designed to distinguish normal (OARSI: 0–1) from general osteoarthritic cartilage (OARSI: 2–5) to check the general suitability of the approach yielding an average accuracy of 75% (AUC = 0.77). Then, the second classifier was designed to distinguish normal from early osteoarthritic cartilage (OARSI: 2–3) yielding an average accuracy of 71% (AUC = 0.73). Important wavelength regions for differentiating normal from early osteoarthritic cartilage were related to collagen organization (wavelength region: 400–600 nm), collagen content (1000–1300 nm) and proteoglycan content (1600–1850 nm). The findings suggest that Vis-NIRS allows objective differentiation of normal and early osteoarthritic tissue, e.g., during arthroscopic repair surgeries.

**Supplementary Information:**

The online version contains supplementary material available at 10.1007/s10439-023-03261-7.

## Introduction

Articular cartilage is a soft tissue that covers the end of bones in the knee joint and enables a near-frictionless joint movement [45]. Osteoarthritis (OA) is a degenerative joint disease that alters the structure and composition (i.e., collagen and proteoglycan content) of cartilage [9, 27]. Early detection of OA is currently not possible, but it would be important to facilitate early opportunities for interventions, such as weight loss or rehabilitation that may slow down OA progression [18, 36]. Yet, early intervention opportunities are often missed as there are no diagnostic methods sensitive to early OA changes.

In laboratories, histological techniques are used to assess osteoarthritic changes in cartilage [33, 49]. However, these histological methods are clinically unsuitable as they require tissue biopsy that is destructive and time-consuming [7]. Also, current OA diagnosis is based on clinical examination, MRI, and radiography [10]. Arthroscopy repair surgeries of joint injuries also present an opportunity to evaluate early degenerative changes in cartilage matrix [10, 16]. While these radiographic techniques and visual arthroscopic methods can enable identification of advanced OA tissue, they are less effective for identifying early OA tissue [6, 7, 24]. This prompts the need for novel methods to detect early degenerative changes in articular cartilage to facilitate early intervention.

Optical spectroscopy, including visible (Vis) light and near-infrared spectroscopy (NIRS), is a group of techniques based on the ability of a material to absorb or scatter light in the electromagnetic spectrum [2, 7]. NIRS combined with multivariate modeling has been previously used to estimate cartilage thickness and instantaneous modulus [40]. Cartilage composition and structure are altered in OA, impacting light scattering and absorption in the tissue, leading to subtle differences in optical spectral properties [19].

Studies on NIRS of cartilage have focused on animal models [3, 29, 38] and on differentiating normal from advanced OA [29] tissue where clinical intervention has minimal impact. In a clinical setting, identifying normal from early OA would be more beneficial, as early diagnosis would enable earlier intervention that could substantially delay or halt disease progression. Previous studies have also not accounted for site-specific properties of cartilage. Site-specific differences [6, 29, 37] in cartilage structure, composition, and mechanical properties have been shown to occur due to different loading conditions in joint sites [23, 30, 44]. This variation might impact the absorption and scattering properties of NIR light in each joint site, especially during arthroscopic procedures. Hence, a generalized optics-based method for differentiating normal from OA tissue may not account for the variation between joint locations.

Due to the overlapping nature of NIR spectral features, nonlinear algorithms have been shown to outperform the more “traditional” principal component analysis (PCA) and partial least discriminant analysis (PLS-DA) [29] in predicting tissue integrity [39]. For example, artificial neural networks [38], logistic regression and support vector machines (SVM)-based nonlinear algorithms have been applied to monitor cartilage integrity [3]. In comparison to other nonlinear methods, SVM can be used on a small dataset [11]. SVM is also more robust and less prone to the curse of dimensionality, which is a significant challenge when dealing with spectral data [5].

This study aims to develop site-specific SVM classifiers to differentiate normal cartilage from early-stage OA cartilage based on their Vis-NIR spectra. We hypothesize that SVM can differentiate normal cartilage from early OA cartilage as Vis-NIRS is sensitive to changes in the cartilage matrix components. Compared to normal cartilage, the biomechanical properties of OA cartilage are reduced significantly [12]. Thus, the SVM classifiers were further validated by comparing tissue biomechanical properties, first, based on OARSI grades assigned by trained researchers before SVM classification, and second, based on the predictions of the SVM classifiers. The findings of the current study should provide support for the incorporation of Vis-NIR spectroscopy into conventional arthroscopy procedures and ex vivo studies for objective and fast assessment of tissue quality.

## Materials and Methods

### Sample Collection

**Fig. 1:**
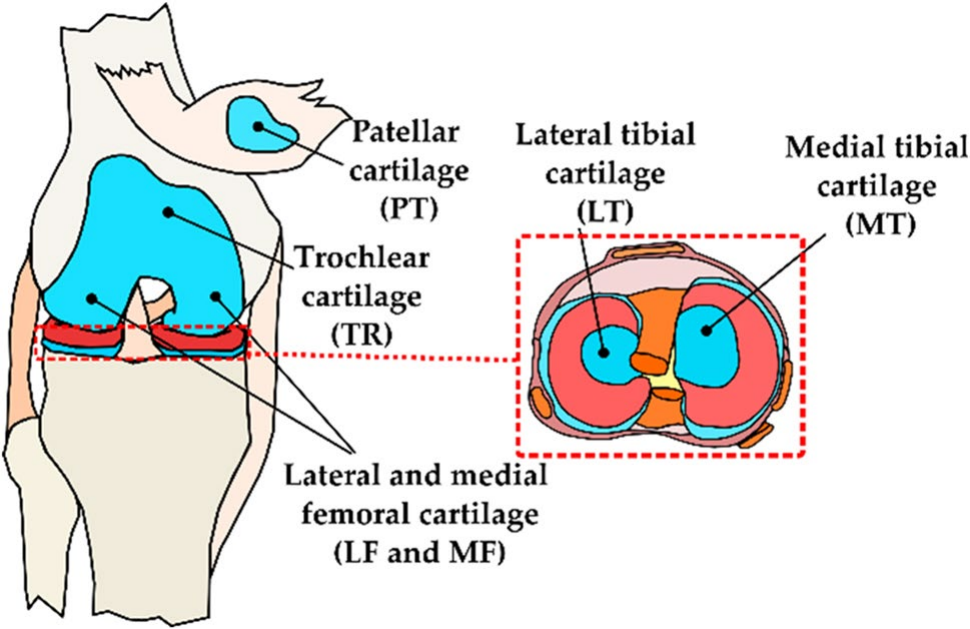
Schematic of the knee map. Cartilage samples were extracted from the patella, trochlea, femur, and tibia regions. Modified from [32]

**Fig. 2:**
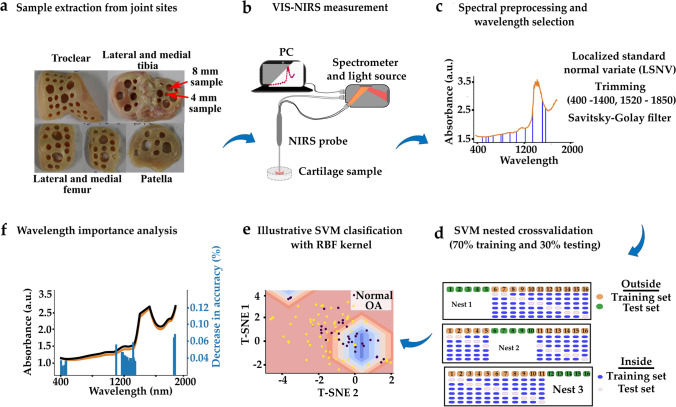
**a** Cartilage samples were extracted from six (6) sites of the human knee joint. **b** The samples were characterized using Vis-NIR spectroscopy followed by biomechanical testing of the samples. Subsequently, histological sections of the samples were prepared and graded according to OARSI grading. **c** The acquired spectra were trimmed (400–1400 nm, 1520–1850 nm) and the 30 most important wavelengths were selected using a sequential feature selection algorithm. **d** This was followed by model training based on SVM. **e** A T-distributed Stochastic Neighboring Entity (t-SNE) plot is used to illustrate SVM classification. The blue concentric diamond-shaped rings show the probability of classification as normal tissue. The purple dots indicate normal tissue, and the yellow dots indicate OA tissue. **f** Permutation feature importance was used to rank wavelength contribution to classifier accuracy

Human cartilage samples [*N* = 179; sample diameter (*d*) = 4 and 8 mm] were obtained from the left and right knee joints of 8 cadaver donors (3 males, 5 females, and age = 64.8 ± 9.12) obtained from a commercial biobank (Science Care, USA). Additional samples (*N* = 161, *d* = 8 mm) were collected from 9 cadaver donors (7 males, 2 females and age = 68.4 ± 7.45 years) obtained from Kuopio University Hospital, Kuopio Finland (Table 1). Full-thickness osteochondral samples (cartilage and bone) were extracted from the lateral and medial tibia (LT and MT), lateral and medial femur (LF and MF), trochlear (TR), and patella (PT) sites (Figs. 1 and 2a) of the knee joint using a 4 mm and 8 mm dental drill (NTI-Kahla Rotary Dental Instruments, Khala, Germany). The samples were drilled perpendicularly to the tissue surface. Then, the samples were immersed in a phosphate-buffered saline solution (PBS) and stored in the freezer at − 20 °C*.* Before testing, the samples were removed from the freezer and thawed at room temperature. The Research Ethics Committee of the Northern Savo Hospital District approved the study (Kuopio University Hospital, Kuopio, Finland, #134//2015).

**Table 1:** Sample demographic (age, sex, OARSI grade, and the number of samples), for cadavers obtained from the commercial biobank and the Kuopio University Hospital

	Serial number	Age	Sex	OARSI 0 samples	OARSI 1 samples	OARSI 2 samples	OARSI 3 samples	OARSI 4 samples	OARSI 5 samples	Total
Biobank cadavers (*n* = 8)	1	70	F	4	3	7	6	3	0	23
	2	62	F	1	4	3	4	6	3	21
	3	64	M	2	3	5	4	8	2	24
	4	47	F	5	6	9	4	1	0	25
	5	71	M	0	3	3	4	8	4	22
	6	68	M	4	4	4	4	8	4	23
	7	74	F	2	3	5	4	4	1	19
	8	63	F	3	1	2	4	12	0	22
Hospital cadavers (n = 9)	1	68	M	3	7	6	5	n/a	n/a	21
	2	68	M	0	5	9	3	n/a	n/a	17
	3	79	F	0	1	4	4	n/a	n/a	9
	4	79	M	1	3	4	4	n/a	n/a	12
	5	68	M	1	6	8	2	n/a	n/a	17
	6	69	M	0	7	18	4	n/a	n/a	29
	7	69	F	0	5	3	1	n/a	n/a	9
	8	59	M	3	3	11	2	n/a	n/a	19
	9	74	M	2	9	11	6	n/a	n/a	28

### Vis-NIR Spectra Measurement

**Fig. 3:**
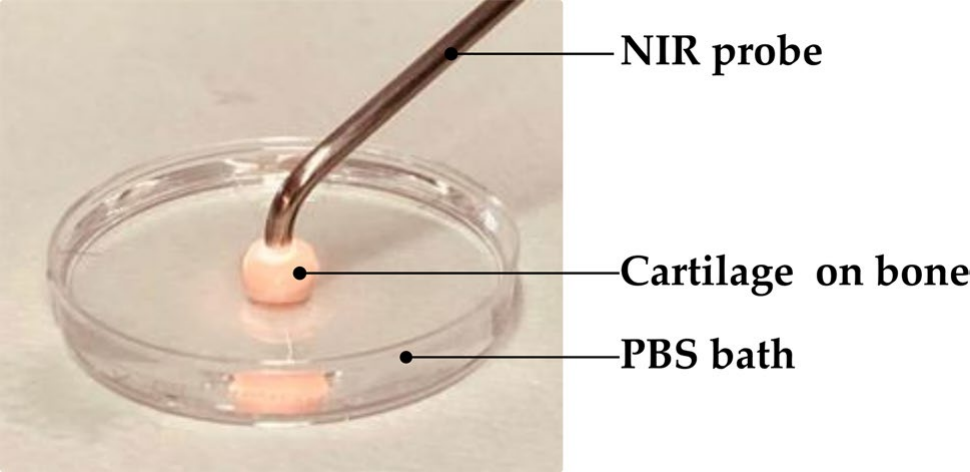
Schematic of the spectral data collection setup

Vis-NIR spectral measurements (*N* = 927) of the cartilage samples were carried out using a NIR system (AvaSpec Multichannel spectrometer, Avantes BV, Apeldoorn, the Netherlands, Fig. 3). The system is composed of two detectors (detector for Vis spectra: AvaSpec-ULS2048L, *λ* = 350–1100 nm and spectral resolution = 0.6 nm; detector for NIR spectra: AvaSpec-NIR256-2.5-HSC, *λ* = 1000–2500 nm, spectral resolution = 6.5 nm) and a 10 W halogen lamp light source. This device has a custom-designed stainless steel arthroscopy probe. The probe (*d* = 3.25 mm) has a 2 mm diameter window at the tip which contains 114 small optical fibers (*d* = 100 µm) and 14 of those are used to collect the scattered and reflected Vis-NIR light. Every collected spectrum was an average of 10 co-added scans with an integration time of 30 ms. Three (3) single-point spectra were collected from the middle of each osteochondral sample.

### Biomechanical Testing and Analysis

**Fig. 4:**
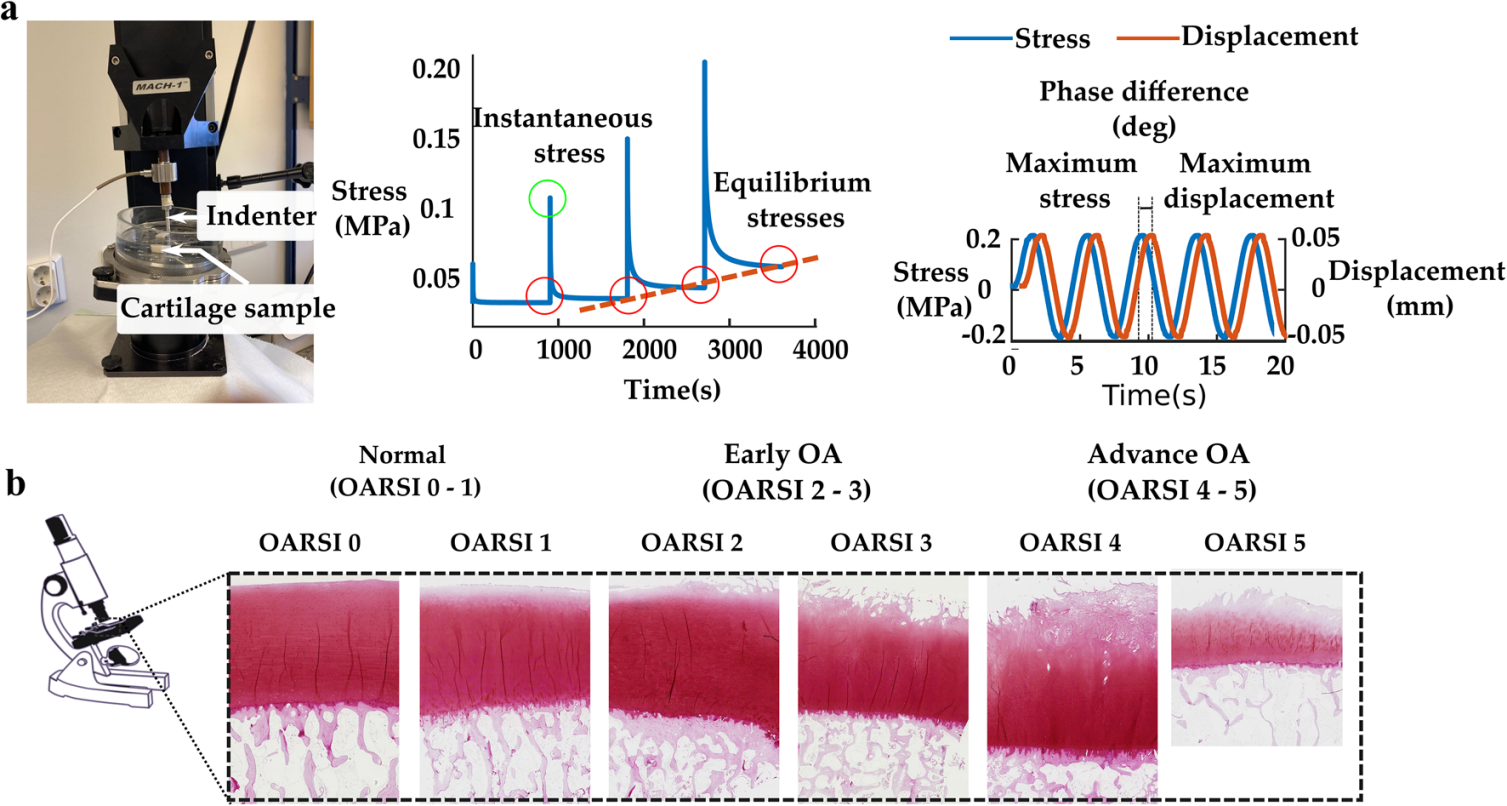
**a** Biomechanical stress-relaxation and dynamic testing were performed. Instantaneous, equilibrium, dynamic moduli and phase angles were determined for each sample. **b** Representative histological images of cartilage samples with varying histological grades.

First, cartilage thicknesses were measured around the circumference of the sample using a stereomicroscope (STEMI, SV8, Zeiss, Germany). Three equally spaced points were measured and averaged to obtain an average thickness for each sample. A biomechanical testing system (MACH-1, Biomomentum Inc., Laval, Quebec, Canada) was used for indentation testing (flat-ended steel indenter, *d* = 1 mm). During the measurement, the samples were immersed in PBS and the measurement chamber was tilted until the individual sample surfaces were perpendicular to the indenter. An equilibrium pre-stress of 12.5 kPa on the cartilage surface was applied to ensure a good contact [12, 27]. Then, a 4-step stress-relaxation protocol was applied. Each step was comprised a 5% compression (of the remaining thickness of each sample) followed by a 15 min relaxation (Fig. 4a). The percentage thickness minimized this impact of thickness variation on the mechanical properties. Immediately after the last step of the stress-relaxation protocol, a four-cycle dynamic sinusoidal test was initiated with a 2% amplitude (of remaining thickness) and frequency of 1 Hz. Equilibrium Young’s modulus was calculated from the slope of the linear least-squares fit to the equilibrium stress values of each stress-relaxation step [8, 12]. Instantaneous modulus was determined at 10% strain. Dynamic modulus and phase difference were calculated as the ratio of the peak stress and strain and the difference in phase lag between stress and strain, respectively. Hayes correction was applied in each moduli calculation to account for the effect of the finite sample thickness and indenter diameter in indentation geometry [15]. The Poisson’s ratio for equilibrium and instantaneous moduli was set to 0.3, and for dynamic modulus was set to 0.5 assuming the tissue was incompressible [12].

### Histopathological Scoring

The samples were first fixed and decalcified in a mixture of formalin and 0.5% EDTA solution and then paraffin embedded. Adjacent 5 µm thick histological sections were cut with a microtome. Subsequently, the sections were stained with Safranin-O and transferred to a microscopy slide. Osteoarthritis Research Society International (OARSI) grading system (Supplementary Table S1) was used to grade the disease severity of the samples [33]. OARSI scores were assigned to each histological section by three trained researchers. The scores from each grader were then averaged and rounded to the nearest integer for the final score of the sample (Fig. 4b). The samples were pooled into normal (OARSI 0–1), early OA (OARSI 2–3), and advanced OA (OARSI 4–5) groups. These groups were adopted to maintain a relatively similar proportion of samples in the normal and OA groups.

### Spectral Data Preprocessing

A custom python-based software, Nippy (available at: https://github.com/uef-bbc/nippy), was used to preprocess the spectra [47]. For preprocessing, Savitzky–Golay filter was used for data smoothing and spectral normalization was performed using localized standard normal variate (LSNV). Due to spectral saturation (noise) around the OH peak (1400–1520 nm), which is indicative of cartilage water content [21], each spectrum was trimmed to the spectral regions of 400–1400 nm and 1520–1850 nm. While the region (1000–1399 nm) has been linked to cartilage collagen content [1, 31], the region (1600–1800 nm) is associated with the matrix proteoglycan content [1, 31]. Wavelength selection was then performed using a forward sequential feature selection (SFS) algorithm (description in supplementary material) to select the optimal wavelengths for classification.

### Support Vector Machine (SVM) Classification

The wavelengths selected via SFS algorithm were applied to the SVMs. SVM takes in a matrix of predictors (e.g., multidimensional spectral data) and targets (e.g., cartilage integrity classes), and attempts to transform the data using algorithms called kernels (e.g., radial, linear, polynomial, and sigmoid) such that the classes are linearly separable. The choice of kernel function is optimized using the “gridsearch” approach whereby each combination of hyperparameters within the kernel functions is evaluated to find the optimal set of parameters that maximizes class separability.

Two different SVM classifiers were trained and tested for the identification of normal and OA tissue. *Classifier 1* was trained to differentiate normal tissue (OARSI 0–1) from OA tissue (OARSI 2–5) and consisted of 256 and 425 spectra, respectively. To minimize bias towards the majority class, a class weight inversely proportional to the number of spectra in each group was used to account for class imbalance. *Classifier 2* was trained to differentiate normal tissue (OARSI 0–1) from early OA tissue (OARSI 2–3) with 256 spectra in each group. Classifier 1 has limited clinical relevance as it considers a wide range of OA (OARSI 2–5), including samples from macroscopically visible OA damage, rather it was designed as a baseline classifier to compare the performance of Classifier 2. The input for SVM was the NIR spectra, and the output was the SVM predictions (normal vs OA in Classifier 1 and normal vs early OA in Classifier 2). To evaluate the effect of wavelength selection, in each joint site, Classifier 1 was also applied to the full wavelength range and the test-set accuracy over the full wavelength range was compared to the accuracy of Classifier 1 when 30 wavelengths were selected.

To train the classifiers the samples were split according to cadavers into a training set (70% of the dataset per site) and a test-set (30% of the dataset per site). Each classifier was trained using a 5-fold cross-validation approach and the model with the highest cross-validation accuracy was then selected and applied to the test-set. This process was repeated three times for each site. To ensure robust training, the data splitting was done so that the training and the test-set included samples and spectra from different cadavers. Permutation feature importance was applied to identify important wavelengths based on the percentage reduction in classifier accuracy when a wavelength is eliminated. SVM classification and wavelength importance analysis was performed in the Scikit-learn package (version 3.8.3) in Python (version 3.8.5). After the classification, the samples were regrouped based on the classifier predictions (i.e., samples classified as normal vs samples classified as OA in Classifier 1 or samples classified as normal vs samples classified as early OA in Classifier 2) also referred to as prediction groups.

### Classifier Evaluation

The elements of a confusion matrix were used to evaluate the classification performances [41]. True positive (TP) refers to OA tissue spectra (early OA or OA) that are correctly classified as OA and False Positive (FP) shows the normal tissue spectra that are falsely classified as OA (early OA or OA). Whereas the False Negative (FN) indicates the OA tissue spectra (early OA or OA) spectra falsely classified as normal tissue and the True Negative (TN) is the spectra of normal tissue classified as normal. The classification performances including sensitivity, specificity, and accuracy are defined based on the TP, FP, FN, and TN of a confusion matrix.

Sensitivity was defined as the ratio of spectra accurately classified as OA (early OA or OA) out of all the OA (early OA or OA) tissue spectra classified:

1 $$\text{Sensitivity}= \frac{\text{TP}}{\text{TP}+\text{FN}}.$$

Specificity was defined as the ratio of spectra accurately classified as normal out of all normal tissue spectra classified:

2 $$\text{Specificity}= \frac{\text{TN}}{\text{TN}+\text{FP}}.$$

Accuracy was defined as the proportion of correctly classified spectra out of the total number of spectra classified:

3 $$\text{Accuracy}= \frac{\text{TP}}{\text{TP}+\text{FP}}.$$

Furthermore, the classifiers were assessed based on the Area Under the Receiver Operating Characteristic (ROC) Curves (AUC). AUC-ROC measures the ability of the classifiers to avoid false classification.

### Statistical Analysis

*Statistical groups*: Three sets of statistical comparisons were made. The first comparison was based on the blindly assigned OARSI groups before SVM classification and compares the biomechanical properties of normal (OARSI 0–1) vs OA (OARSI 2–5) tissue used in Classifier 1 and normal (OARSI 0–1) vs early OA (OARSI 2–3) tissue used in Classifier 2. The second comparison was based on the SVM prediction groups and aimed to validate the classifiers. The comparison was between samples classified as normal vs OA (OARSI 2–5) in Classifier 1 and samples classified as normal vs early OA (OARSI 2–3) in Classifier 2. Lastly, comparison was made between the biomechanical properties of normal, early OA and advanced OA groups based on the blindly assigned OARSI groups.

A linear mixed model was used for statistical analysis since multiple samples were collected per cadaver. The linear mixed effect model was chosen because it can account for interdependencies between the samples from the same cadaver [26]. In the models, cadavers (subjects) were set as the random effect variables while the OARSI groups were set as fixed effect variables [26]. Statistical significance was set at *p* < 0.05. Fisher's least significant difference (LSD) posthoc analysis was used to obtain estimates. All statistical analyses were performed with IBM SPSS Statistics (Version 25, IBM Corporation, Armonk, NY, USA).

## Results

### Mean Vis-NIRS Spectrum and Effect of Wavelength Selection

**Fig. 5:**
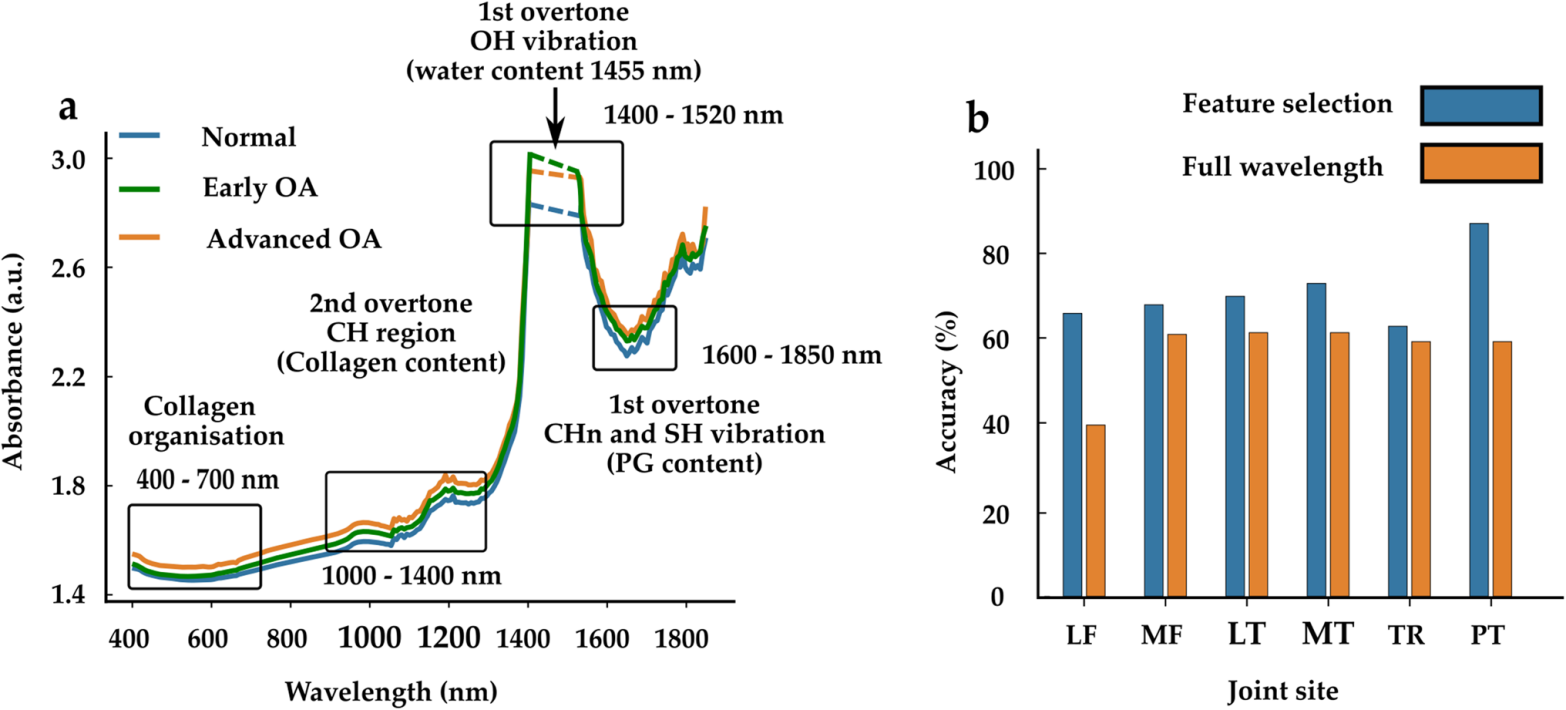
**a** The mean spectra comparison of normal, early OA, and advanced OA tissue. Spectral regions associated with collagen structure (400–700 nm) [17] based on scattering, collagen content (1000–1400 nm) [1, 31], and proteoglycan content (1600–1800 nm) [1, 31] are indicated. Due to noise, the spectral region 1400–1520 nm and 1850–2400 nm were truncated, **b** Site-specific comparison of model accuracy when full spectral wavelengths are included and when 30 wavelengths are selected using sequential feature selection.

The mean spectra showed slight differences between normal, early OA, and advanced OA tissue (Fig. 5a). Differences were observed in the first overtone CH vibrations estimated region 1130–1333 nm [33, 34] and 1156 nm peak [13], the CH_n_ and SH vibrations estimated region (1600–1800 nm) [33]. Differences in baseline offset at the Vis region (400–700 nm) [17] were also observed between the groups. The effect of wavelength selection on the classification accuracy is presented in Fig. 5b. In all sites, the classification accuracies increased when using the selected 30 wavelengths compared to the full spectrum. The highest improvement in accuracy was in the patellar (PT) and lateral femur (LT) sites with 28% and 26% increases, respectively. The least accuracy increase was in the trochlear site with a 4% accuracy increase.

### Classifier Performances

**Fig. 6:**
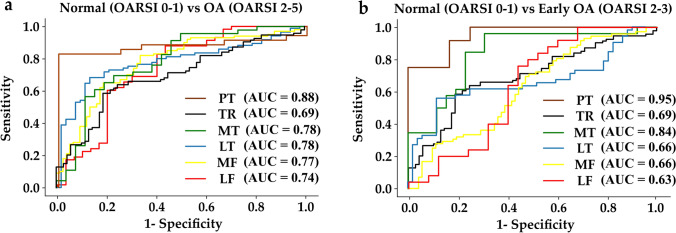
ROC curves and corresponding areas under the curve (AUC) for evaluating the ability of Vis-NIR to detect normal from OA or early OA tissue based on OARSI groups in each joint site, **a** AUC for Classifier 1 and, **b** AUC for Classifier 2. AUC measures the ability of the classifiers to avoid erroneous classification.

Classifier 1 (classification of normal versus OA cartilage): The patellar site had the highest classification accuracy of 87.0% with an AUC of 0.88 (Fig. 6a), while the trochlear region had the lowest accuracy of 69.0% with an AUC of 0.69 (Fig. 6a), The average accuracy across all sites was 75% with an average AUC = 0.77. The average sensitivity and specificity were 0.74 and 0.75 (Table 2).

**Table 2:** Test-set accuracies, sensitivity, and specificity of Classifier 1 and Classifier 2. Classifier 1 compares normal (OARSI: 0–1) and OA (OARSI: 2–5) cartilage and Classifier 2 compares normal (OARSI: 0–1) and early OA (OARSI: 2–3). *LF* lateral femur, *MF* medial femur, *LT* lateral tibia, *MT* medial tibial, *TR* trochlear, *PT* patellar

	Classifier 1			Classifier 2		
Location	Accuracy	Sensitivity	Specificity	Accuracy	Sensitivity	Specificity
LF	0.72	0.57	0.81	0.66	0.80	0.52
MF	0.70	0.88	0.57	0.68	0.64	0.56
MT	0.77	0.64	0.88	0.70	0.69	0.77
LT	0.71	0.89	0.54	0.73	0.56	0.85
TR	0.69	0.70	0.70	0.64	0.45	0.83
PT	0.88	0.77	1.00	0.87	0.92	0.83

Classifier 2 (classification of normal versus early OA cartilage): the patellar site had the highest accuracy of 87.0% with an AUC of 0.95 (Fig. 6b), while the trochlear site had the lowest accuracy of 64% with an AUC of 0.69 (Fig. 6b). The average accuracy across all regions was 71% which was only 4 percentage points smaller compared to Classifier 1. The average specificity and sensitivity were 0.67 and 0.72 (Table 2).

### Wavelength Importance Analysis

In Classifier 1, the wavelength regions (400–600 nm), (1000–1400 nm), and (900–1300 nm) contributed most to the model accuracy (Supplementary material Fig S3). In Classifier 2, the wavelength regions (400–700 nm), (900–1200 nm), and (1600–1800 nm) contributed most to the model accuracy (Supplementary material Fig S4).

### Biomechanical Properties of the OARSI Groups and the SVM Prediction Groups

**Fig. 7:**
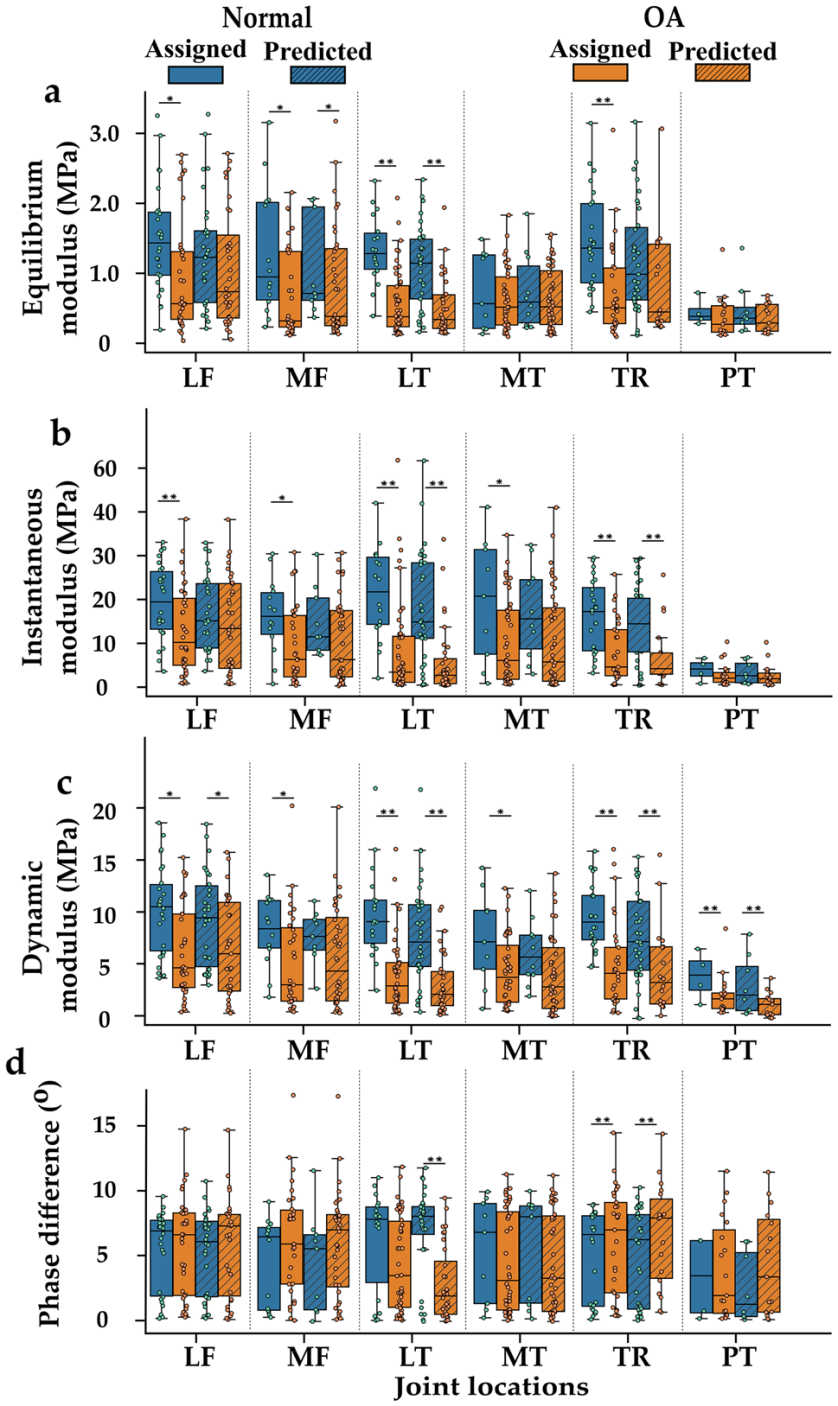
Comparison between normal and OA tissue based on OARSI groups assigned by trained individuals (normal = OARSI 0–1 and OA = OARSI 2–5) and groups assigned based on Classifier 1 predictions. The plain box and whisker plots show the biomechanical properties of OARSI groups. The box with oblique lines shows the biomechanical properties of Classifier 1 prediction groups. The dynamic modulus and phase angles were analyzed at 1 Hz loading frequency. *LF* lateral femur, *MF* medial femur, *LT* lateral tibial, *MT* medial tibial, *TR* trochlear, *PT* patella. *p < 0.05 and **p < 0.001

*Classifier 1*: when comparing biomechanical properties based on the OARSI groups (normal = OARSI 0–1 and OA = OARSI 2–5) the dynamic and instantaneous modulus were significantly higher in normal tissue compared to OA tissue across the joint sites (*p* < 0.01 and *p* < 0.05 depending on the site) except for the patellar cartilage (*p* > 0.05). The equilibrium modulus was significantly higher in normal tissue compared to OA tissue (*p* < 0.01 and *p* < 0.05 depending on the site), except for the medial tibial and patellar cartilage (*p* > 0.05) (Fig. 7). Table 3 shows the mean biomechanical properties of the groups. When the samples were grouped based on the SVM classification of Classifier 1 (normal and OA), similar differences in biomechanical properties between tissue classified as normal and tissue classified as OA tissue were observed; although, the difference was not always statistically significant.

**Table 3:** Mean [95% confidence intervals] of biomechanical properties of normal, early OA and advanced OA groups

Parameter	Normal (OARSI 0–1)	Early OA (OARSI 2–3)	Advanced OA (OARSI 4–5)
*E*_eq_ (MPa)	1.29 [1.15, 1.44]	0.74 [0.65, 0.83]^a^	0.164 [0.12, 0.20]^a,b,c^
*E*_inst_ (MPa)	19.12 [17.04, 21.20]	11.57 [10.15, 12.99]^a^	1.67 [1.19, 2.15]^a,b,c^
*E*_dyn_ (Mpa) at 1 Hz	9.42 [8.62, 10.22]	5.46 [4.89, 6.02]^a^	1.517 [1.13, 1.90]^a,b,c^
Phase angle (^o^) at 1 Hz	5.37 [4.72, 6.02]	6.12 [5.54, 6.70]	2.88 [2.13, 3.63]^a,b,c^
Thickness (mm)	2.37 [2.28, 2.46]	2.57 [2.47, 2.68]^a^	2.27 [2.08, 2.46]^b^

E_eq_: Equilibrium modulus, E_inst_: instantaneous modulus, E_dyn_: dynamic modulus

^a^Significant difference (*p* < 0.05) to the normal group

^b^Significant difference (*p* < 0.05) to the early OA group

^c^Significant difference (*p* < 0.05) between the normal and advanced OA groups

**Fig. 8:**
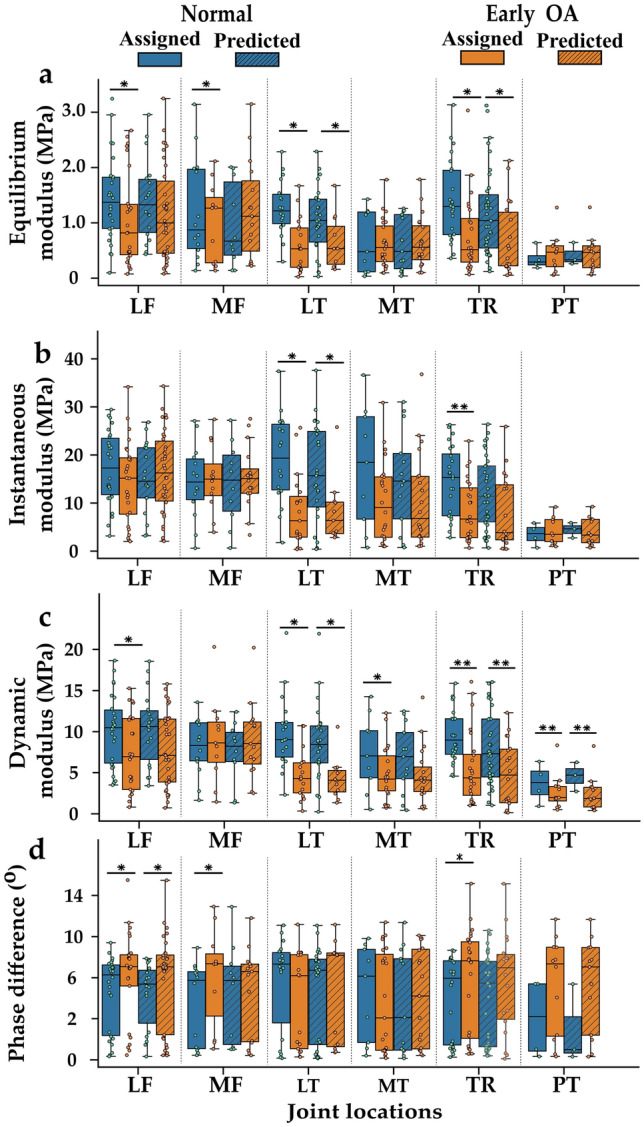
Comparison between normal and OA tissue based on OARSI groups assigned by trained individuals (normal = OARSI 0–1 and early OA = OARSI 2−3) and groups assigned based on Classifier 2 predictions. The plain box and whisker plots show the biomechanical properties of OARSI groups. The box with oblique lines shows the biomechanical properties of Classifier 2 prediction groups. The dynamic modulus and phase angles were analyzed at 1 Hz loading frequency. *LF* lateral femur, *MF* medial femur, *LT* lateral tibial, *MT* medial tibial, *TR* trochlear, *PT* patella. *p < 0.05 and **p < 0.001

Classifier 2: when comparing the biomechanical properties of the OARSI groups assigned by trained individuals the equilibrium modulus of normal cartilage was significantly higher than early OA cartilage in all sites (*p* < 0.001 and *p* < 0.05 depending on the site), except in patella and medial tibia (*p* > 0.05) (Fig. 8). The instantaneous modulus of the normal cartilage was significantly higher compared to early OA cartilage in the trochlear (*p* < 0.001) and the lateral tibia (*p* < 0.05) sites. The dynamic modulus was significantly higher for normal cartilage compared early OA cartilage in all sites (*p* < 0.001 and *p* < 0.05 depending on the site), except in the patella and the medial femur (*p* > 0.05). When the samples were grouped based on the classifications of the classifier, equilibrium, instantaneous and dynamic modulus of cartilage classified as normal were higher compared to cartilage classified as early OA (though, the difference was not always statistically significant).

## Discussion

In this work, Vis-NIRS combined with SVM algorithm was used to detect normal cartilage from osteoarthritic human knee cartilage in a site-specific manner. SVM classifiers trained on the Vis-NIRS spectral data were able to distinguish normal from OA tissue (Classifier 1) and normal tissue from early OA tissue (Classifier 2). The classes (normal, early OA, and “general” OA tissue) were established based on histopathological OARSI grading blindly assigned by trained researchers. The classifiers were further validated by evaluating the tissue biomechanical properties based on the assigned OARSI groups and by evaluating the biomechanical properties based on the predictions of the classifiers. As we hypothesized, Vis-NIRS combined with SVM was able to distinguish normal from early OA tissue.

### Vis-NIR Classification of Normal Versus OA (Classifier 1) and Normal Versus Early OA Cartilage (Classifier 2)

In the study, the average accuracy of Classifier 1 was 75% (AUC = 0.77) and decreased to 71% (AUC = 0.73) in Classifier 2. The decrease in classification accuracy is likely due to a much larger variation in tissue structure and composition between the normal (OARSI 0-1) and OA (OARSI 2–5) groups (Classifier 1) compared to the normal (OARSI 0−1) and early OA (OARSI 2−3) group (Classifier 2) where the variation is smaller. This was verified via a feature importance analysis that showed similar spectral regions that contributed the most to the classification accuracy in both classifiers. During cartilage degeneration, water content increases, PG decreases, and collagen loses orientation [28]. While the increased water content and reduced PG alter tissue light absorption, collagen disorientation affects the light scattering and absorption profiles [19]. The variation in light absorption due to the spectrum of these properties from normal to early OA and advanced OA might have influenced the classifier performances. The highest classification accuracy was observed in the patellar location. This might be attributed to material density and stiffness, since the spectral baseline in NIR diffuse reflectance spectroscopy has been demonstrated to increase with material density as denser materials allow less light to reach the detector [35]. Because the patellar cartilage was softer than the other sites, more photons may reach the detector, improving classification accuracy.

To validate the classifiers, the tissue mechanical properties were compared based on the OARSI groups [normal (OARSI 0–1), early OA (OARSI 2–3), OARSI (4–5)] and based on the predictions of the classifiers (classified normal or classified early OA or OA). In both grouping systems, Classifier 1 showed better separation of normal from OA cartilage compared to Classifier 2. This could also be because absorption and scattering of VIS-NIR light may be less impacted in the early OA tissue compared to the advanced OA tissue, which is associated with significant changes in tissue mechanical properties [33]. However, when making direct conclusions related to model performances (Classifier 2) in assessing early changes in tissue mechanical properties, it should be considered that OARSI grading was used to group samples into normal versus OA groups. This may slightly distort the conclusions, since it is known that material properties in normal tissues may be highly dependent on tissue adaptation, i.e., lifestyle (physical activity level, age, weight) that may influence the tissue biomechanical properties [13, 48].

### Relation of Vis-NIR Spectra to Tissue Composition, Biomechanical Properties, and Site-Specificity

The most informative spectral regions for differentiating normal from degenerated (OA or early OA) tissue were: 400–800 nm (indicating light scattering in tissue) [17], 900–1300 nm (absorption by collagen); 1600–1850 nm (absorption by PG content) [1, 31] (Figs. S3 and S4). In both classifiers, the accuracies in discriminating between normal and degenerated tissue samples were also site-specific. Cartilage site-specific variation in structure, composition, and mechanical properties may result from the different joint mechanical environments [25, 27]. This variation placed some difficulties when interpreting the data as one.

Though equilibrium, instantaneous and dynamic moduli were smaller in advanced and early OA compared to normal tissue, the tissue thickness increased in early OA possibly due to swelling that has been reported to occur in early OA cartilage [4]. In advanced OA, the cartilage thickness was smaller than in early OA, which may be a sign of cartilage erosion [33]. When comparing the biomechanical properties, in Classifier 1, the dynamic and instantaneous moduli of normal cartilage (OARSI 0–1) were significantly different from OA cartilage (OARSI 2–5) across most joint sites (Fig. 7). When the biomechanical properties were grouped based on Classifier 1 predictions (normal or OA), significant differences were still observed in the dynamic modulus of the lateral femur, tibia, and trochlear sites. In cartilage, dynamic and instantaneous stiffness is closely associated with the structure and concentration of tissue collagen. Thus, wavelength importance analysis for Classifier 1 showed that the spectral regions responsible for the classification of normal and OA tissue are consistent with absorption regions related to cartilage collagen content (1200–1400 nm) [1, 31]. Kendal et al. [14, 20] have shown that an increasing baseline offset of Vis-NIR spectra reflects matrix maturation in engineered tissue construct. Since significant scattering has been shown in the visible region [17], the baseline offset in the region (400–600 nm) may reflect a difference in collagen organization between normal and degenerated tissue. In Classifier 2, in the trochlear site, the dynamic and equilibrium properties were significantly different in normal and early OA tissue for both the OARSI groups and prediction groups. Cartilage equilibrium modulus is often linked with tissue PG content and we also found that the wavelength region associated with PG content (1600–1850 nm) [31] was also important for differentiating normal from early OA tissue.

### Vis-NIR as a Tool for OA Assessment

The International Cartilage Repair Society (ICRS) grading is currently used in staging OA during conventional arthroscopy. In conventional arthroscopy, surgeons identify cartilage damage based on visual inspection and manual palpation of the tissue matrix [42, 43]. However, evaluation of early OA is still a challenging task with the ICRS system [30] as early signs of degeneration are not visually apparent and ICRS grading is not originally designed for the quantification of different OA states. Vis-NIRS could be incorporated into an arthroscope to assess cartilage quality based on the interaction of light with cartilage during arthroscopic repair surgery without the need for biopsy samples. While there is currently no comparative study between ICRS and OARSI grades of human tissue, Vis-NIRS offers the advantage of objective and repeatable assessment of early-stage cartilage changes during arthroscopic repair surgery. In clinical settings, absolute values in the peak-specific analysis are likely to yield sub-optimal predictions due to the overlapping and non-specific nature of NIR peaks of cartilage constituents in the Vis-NIR range [21, 34]. Models (e.g., SVM) trained on preprocessed (broadband) spectra of healthy and deteriorated tissue are more likely to evaluate tissue integrity effectively. A potential future direction could be to create a model that converts this information from different wavelengths into an absolute diagnostic value. Further, Vis-NIRS can also be applied to evaluate the development of engineered cartilage tissue to assess implantation readiness [20].

### Limitations

One limitation of the present study is the use of OARSI grading, which assesses tissue pathology based on structural features and stain intensity [33], as a reference for tissue integrity classification. While OARSI grade reflects tissue structural integrity, it is subjective and may poorly represent the tissue compositional changes (PG loss and collagen damage), especially in early OA [18]. Including changes in tissue PG and collagen content may better represent tissue degenerative state and improve the classifier performance. Another limitation is the class imbalance due to difficulty obtaining healthy human cartilage. In classifier 1, the proportion of OA spectra was 74%, 62%, 63%, 58%, 65%, and 74% of the total spectra (normal and OA) in the LF, LT, MT, MF, TR, and PT locations, respectively. If not accounted for, this imbalance may lead to biased performance compared to Classifier 2 with evenly balanced training data. Therefore, we aimed to minimize the potential bias from class imbalance using the class weighting approach, which allows the model to penalize classes with more samples [22]. We also used balanced accuracy, specificity, and sensitivity to evaluate classifier performance. These measures are independent of the class proportions [46]. Also, the 1st overtone OH absorption peak at 1450 nm (within the spectral region 1400–1550 nm) associated with tissue water content was excluded from the analysis due to spectral saturation from the high tissue water content. However, cartilage hydration is a consequence of collagen network damage and PG depletion, hence this parameter could have been captured by the collagen and PG-associated spectral regions. Water and joint fluid have also been shown to be good absorbers of NIR light [38, 39] which can result in spectral saturation if not controlled. To minimize the impact of water, we placed the Vis-NIRS probe in good contact with the cartilage surface. For in vivo measurements where maintaining probe contact may be difficult, we have also developed a method to detect spectra with suboptimal contact [39, 40].

## Conclusion

SVM classifiers developed based on NIRS spectra and OARSI grades were able to differentiate normal from general OA and early OA tissue with reasonable accuracies. Based on a wavelength importance analysis, wavelength regions relating to cartilage proteoglycan content, collagen content, and collagen organization and, consequently, tissue biomechanical properties were important in discriminating between normal and early OA tissue. Vis-NIRS-SVM classification performances show its potential to be incorporated into existing arthroscopy routines for assessing early OA tissue during surgical intervention.

### Supplementary Information

Below is the link to the electronic supplementary material.

Supplementary file1 (PDF 877 KB)
