# Supplementary material for: Visible and Near-Infrared Spectroscopy Enables Differentiation of Normal and Early Osteoarthritic Human Knee Joint Articular Cartilage
Source: Ann Biomed Eng. 2023 Jun 18;51(10):2245–57. doi: 10.1007/s10439-023-03261-7 (PMC10518273; doi:10.1007/s10439-023-03261-7)
Supplement: Supplementary file 1 — Supplementary file1 (PDF 877 KB) [file 10439_2023_3261_MOESM1_ESM.pdf]

Supplementary material for:

Visible and Near-Infrared Spectroscopy Enables Differentiation of Normal and Early Osteoarthritic Human Knee Joint Articular Cartilage

Materials and Methods

Histological grading

Table S 1: OARSI histopathological grading system as described by Pritzker et al [4]. Major degenerative features and associated assessment criteria for various grades.

S/No	Grade	Key features	Description
1	Grade 0	Surface intact cartilage intact	Matrix: Normal matrix architecture. Cells: intact and appropriate orientation.
2	Grade 1	Surface intact	Matrix: intact superficial zone, oedema, superficial fibrillation, focal superficial matrix condensation. Cells: death, proliferation, hypertrophy.
3	Grade 2	Surface discontinuity	As in grade 1. Superficial matrix discontinuity (deep fibrillation). Cationic stain depletion in upper 1/3 of cartilage. Disorientation of chondrons.<br>Cell: death proliferation and hypertrophy.
4	Grade 3	Vertical fissures	As in grade 2. Formation of matrix vertical and branched fissures into the mid zone. Cationic stain depletion into 2/3 of the cartilage. New collagen formation.<br>Cell death, regeneration, and hypertrophy.
5	Grade 4	Erosion	Cartilage matrix loss. Delamination of the superficial layer, cyst formation in the mid-layer. Excavation: matrix degeneration in the superficial and mid-layer.
6	Grade 5	Denudation	Formation of sclerotic bone or reparative tissue. Microfracture with fibrocartilage repair tissue on the bone surface.

7	Grade 6	Deformation	Bone remodeling. Includes formation of microfracture and fibrocartilaginous repair that extends above the previous surface.

Data characteristics

The cartilage samples were collected from 6 different sites (lateral and medial tibia, lateral and medial femur trochlea, patella) of the human knee joint of two different sets of cadaver donors (cadavers from a commercial biobank and the Kuopio University Hospital).

The first set of donors obtained from a commercial biobank comprises samples within the range of OARSI 0 – 5. The second dataset acquired from the Kuopio university hospital comprise samples within the range OARSI 0 – 3 and was added to compensate for the limited number of normal (OARSI 0 – 1) samples in the biobank dataset. When combined dataset formed a nearly normal distribution ensuring the classifiers were trained on a wide range of tissue conditions.

Sample grouping

The samples were graded using the Osteoarthritis Research Society International (OARSI) grading system (Supplementary Table S 1)[28] . Three trained researchers independently assigned OARSI scores to histological section of each sample. The scores from each grader were then averaged and rounded to the nearest integer for the final score of the sample. The samples were pooled into normal (OARSI 0–1), early OA (OARSI 2–3), and advanced OA (OARSI 4–5) groups.s

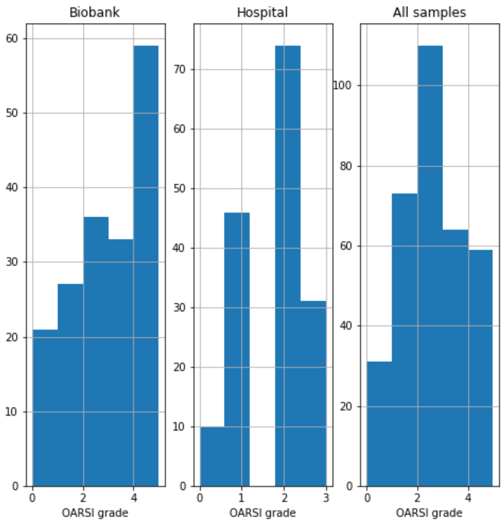

Figure S1: Sample distribution based on OARSI grade from the commercial biobank and the hospital cadavers.

SVM data preparation.

After the removal of noisy spectra and ensuring all 3 spectra from a single sample are either in the training or test set a reference dataset of 681 spectra (400 nm – 1850 nm) across the varying degree of tissue condition and anatomical location in the joint was constructed. Table S2 shows the distribution of data across the normal early OA and OA classes and knee sites.

Table S2: Distribution of spectra based on OARSI grades in the respective joint sites based on OARSI grades. Abbreviations represent (LF: lateral femur, LT: lateral tibial, MT: medial tibial, MF: medial femur, TR: trochlear, PT: patellar)

Sites	Normal (OARSI 0–1)	Early OA (OARSI 2–3)	OA (OARSI 2–5)
LF	25	25	63
LT	52	52	85
MT	26	26	46
MF	72	72	102
TR	69	69	94
PT	12	12	35

Wavelength selection

A forward sequential feature selection (SFS) analysis was performed using mlxtend package in python (version 3.8.5) to select the optimal wavelengths for improved classification accuracy. Forward SFS begins with an empty feature set, an empty wavelength set in our case, and iteratively adds wavelengths that maximize a defined objective function (i.e., wavelengths that result in the highest classifier accuracy) until a predefined number of wavelengths is attained [1,3,5]. Each iteration optimizes an objective function minimizing the performance difference before and after a feature is added:

$$x^{+} = argmax J(X_k + x)$$

Where the feature subset is X_k and x^{+} is the feature that maximizes the objective function (i.e., the feature associated with the best classifier performance). To evaluate the performance in every iteration KNeighbors Classifier (number of neighbors = 3) was used in conjunction with five-fold cross-validation. The number of wavelengths was set to 30. A preliminary assessment showed that this was sufficient and additional wavelengths did not lead to improved model accuracy Fig S2.

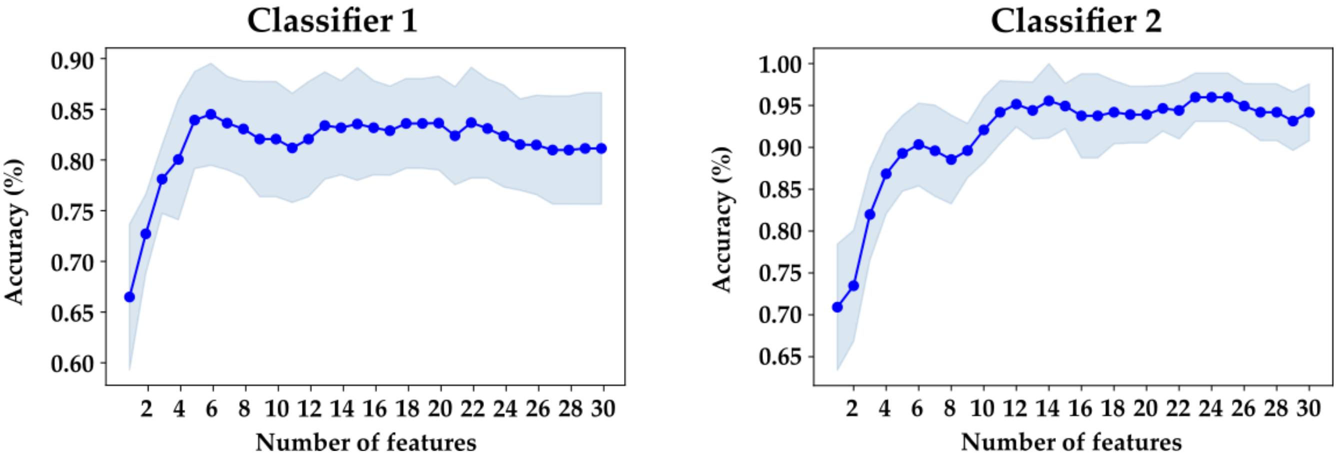

Fig S2: Model performance with iterative feature addition. For every iteration the wavelengths are evaluated using KNeighbors Classifier (number of neighbors = 3). 30 optimal wavelengths were selected for Classifiers 1 and 2.

Parameters optimized in the Support Vector Machines

Depending on the kernel function, the parameters vary but in general include a penalty parameter (*C*), gamma (γ), and polynomial degree [2]. The best classifier is trained based on the set of hyperparameters that yield the highest classification accuracy.

Results

Table S1. Optimal preprocessing and SVM model parameters for classifiers 1 and 2. All spectra were filtered with a Savitsky-Golay filter of parameters Localized standard normal variate (LSNV) preprocessing was used to eliminate noise, then the model was trained using SVM.

Site	Classifier 1			Classifier 2		
	LSNV	Svg filter parameters Derivative(d)/ Window(w)/ Polynomial(p)	SVM parameters C/Degree/ Gamma/Kernel	LSNV	Svg filter parameters Derivative(d)/ Window(w)/ Polynomial(p)	SVM parameters C/Degree/ Gamma/Kernel
LF	12	0/13/1	1/1/scale/rbf	12	None	1/1/scale/linear
MF	12	2/5/2	1/1/0.01/rbf	None	2/5/2	1/1/scale/rbf
LT	12	0/29/2	1/1/scale/rbf	None	2/9/2	1/1/scale/rbf
MT	21	0/9/1	1/1/scale/rbf	4	1/11/1	0.1/1/scale/linear
TR	None	0/13/2	0.1/1/scale/linear	12	1/11/2	1/1/scale/poly
PT	12	0/29/1	1/1/scale/rbf	12	2/9/2	1/1/scale/linear

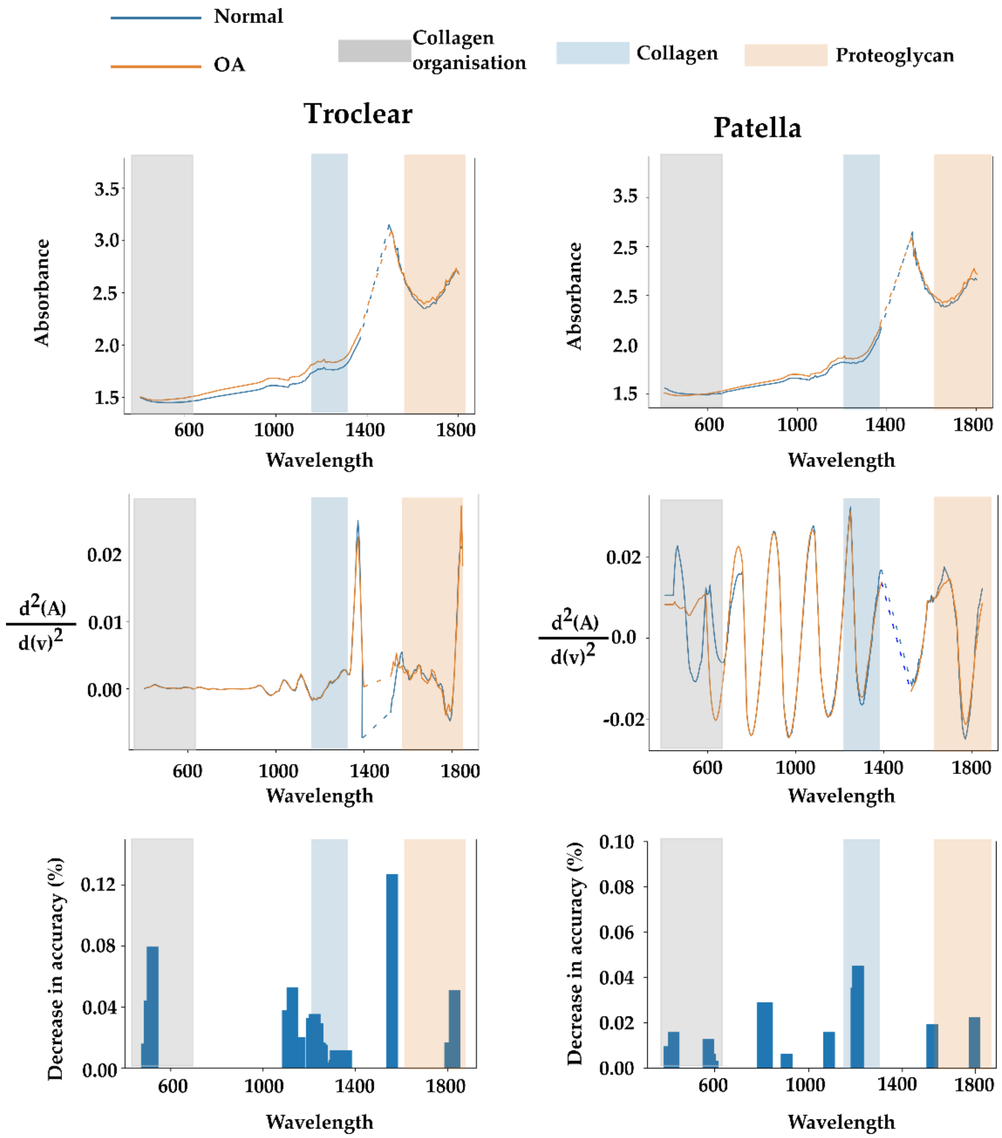

Fig S3: Mean absorption spectra, mean second derivative spectra, and optimal wavelengths for differentiating normal from OA tissue. Representative data are shown for locations with minimum and maximum accuracy in classifier 1. a) Absorptions spectrum of normal and OA tissue b) Second derivative spectra of normal and OA tissue highlighting the difference between the groups c) Important wavelengths for differentiating normal from OA tissue. Wavelength importance was calculated based on permutation feature importance. The height of each bar signifies the mean decrease in model accuracy when the wavelength is randomly shuffled to break the relationship between the wavelength and the group (normal or OA) compared to a baseline. The dash lines showing the water peak (1450 nm – 1520 nm) which was excluded.

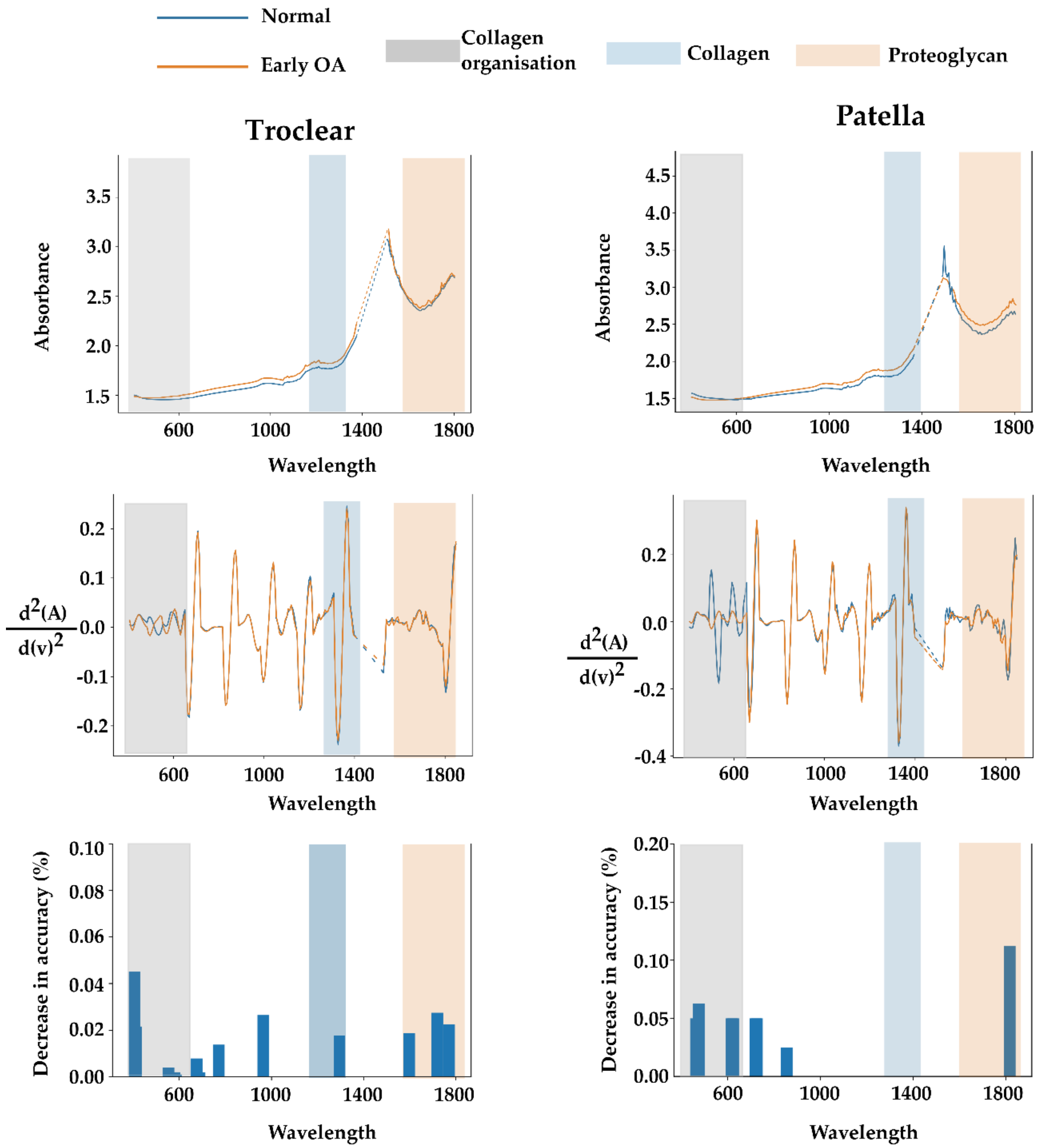

Fig S4: Mean absorption spectra, mean second derivative spectra, and optimal wavelengths for differentiating normal from early OA tissue. Representative data are shown for locations with minimum and maximum accuracy in classifier 2. a) Absorptions spectrum of normal and early OA tissue b) Second derivative spectra of normal and early OA tissue highlighting the difference between the groups c) Important wavelengths for differentiating normal from early OA tissue. Wavelength importance was calculated based on permutation feature importance. The height of each bar signifies the mean decrease in model accuracy when the wavelength is randomly shuffled to break the relationship between the wavelength and the group (normal or early OA) compared to a baseline. The dash lines showing the water peak (1450 nm – 1520 nm) which was excluded.

References

1. Bemister-Buffington, J., A. J. Wolf, S. Raschka, and L. A. Kuhn. Machine learning to identify flexibility signatures of class a GPCR inhibition. *Biomolecules* 10:1–22, 2020.

2. Devos, O., C. Ruckebusch, A. Durand, L. Duponchel, and J. P. Huvenne. Support vector machines (SVM) in near infrared (NIR) spectroscopy: Focus on parameters optimization and model interpretation. *Chemometrics and Intelligent Laboratory Systems* 96:27–33, 2009.

3. Moghimi, P., K. O. Lim, and T. I. Netoff. Data driven classification using fmri network measures: Application to schizophrenia. *Front Neuroinform* 12:1–17, 2018.

4. Pritzker, K. P. H., S. Gay, S. A. Jimenez, K. Ostergaard, J. P. Pelletier, K. Revell, D. Salter, and W. B. van den Berg. Osteoarthritis cartilage histopathology: Grading and staging. *Osteoarthritis Cartilage* 14:13–29, 2006.

5. Singh, K. P., N. Basant, and S. Gupta. Support vector machines in water quality management. *Anal Chim Acta* 703:152–162, 2011.
